# Different anatomical variations in the anterior segment of the right upper lung

**DOI:** 10.1186/s13019-024-02679-x

**Published:** 2024-04-16

**Authors:** Wu Yan, MengXuan Wang, JiXing Zhao, YongSheng Li, Wei Wei

**Affiliations:** 1 Department of Thoracic Surgery, Huizhou Central People’s Hospital, Huizhou, GuangDong China; 2 Department of Medical Imaging, Huizhou Hospital for Occupational Disease Prevention and Treatment, Huizhou, GuangDong China

**Keywords:** Video-assisted thoracoscopic surgery (VATS), Segmentectomy, Three-dimensional reconstruction

## Abstract

During a routine physical examination three years ago, a 47-year-old woman received a diagnosis of a nodule in her right upper lung. Since then, she has been regularly attending outpatient clinic appointments for follow-up. Over time, the nodule has shown gradual growth, leading to a suspicion of lung cancer. Through the use of enhanced CT imaging, a three-dimensional reconstruction was performed to examine the bronchi and blood vessels in the patient’s chest. This reconstruction revealed several variations in the anatomy of the anterior segment of the right upper lobe. Specifically, the anterior segmental bronchus (B3) was found to have originated from the right middle lung bronchus. Additionally, the medial subsegmental artery of the anterior segmental artery (A3b) and the medial segmental artery (A5) were observed to share a common trunk. As for the lateral subsegmental artery of the anterior segmental artery (A3a), it was found to have originated from the right inferior pulmonary trunk. Furthermore, the apical subsegmental artery of the apical segmental artery (A1a) and the posterior segmental artery (A2) were found to have a shared trunk.

## Introduction

Segmental pneumonectomy is considered the recommended surgical approach for patients with small peripheral non-small cell lung cancer [1]. Prior to the operation, it is crucial for thoracic surgeons to have a thorough understanding of the anatomical structure of the pulmonary segment. Minics three-dimensional reconstruction technology can be employed to reconstruct the bronchi and blood vessels from chest CT images, enabling surgeons to visualize the anatomical structure and variations of lung segments from multiple perspectives. As a result, performing anatomical segmental pneumonectomy and conducting three-dimensional reconstruction of blood vessels and trachea prior to the operation becomes highly necessary.

### Case

**Fig. 1:**
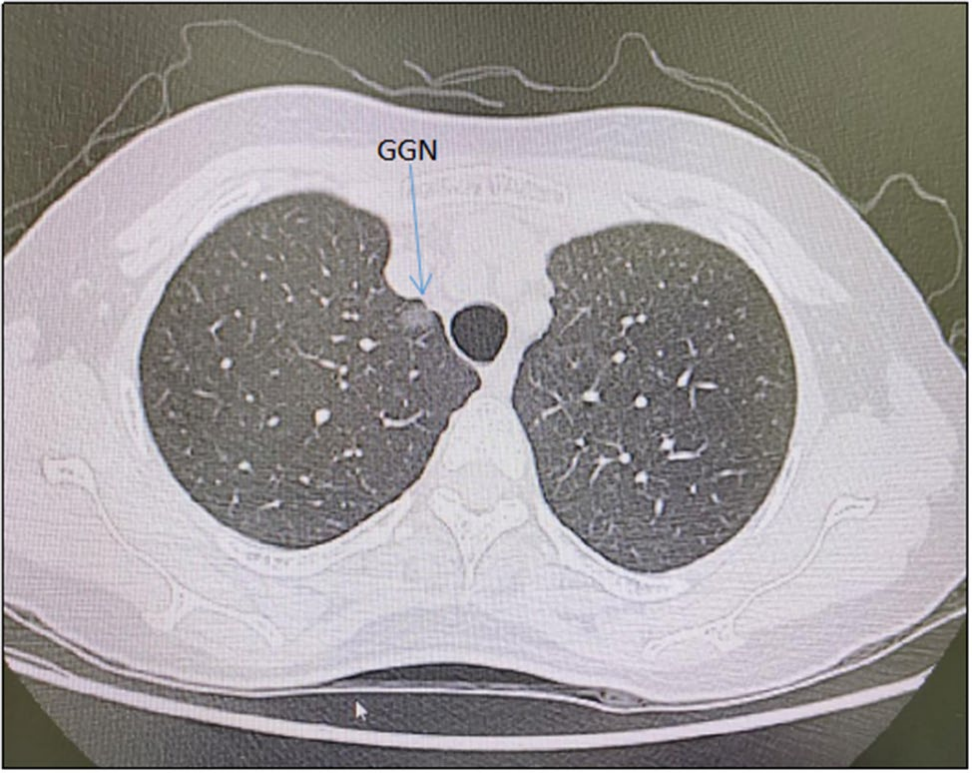
The preoperative chest computed tomography (CT) scan revealed a ground glass nodule (GGN) measuring 10 mm in diameter, located near the pleura of the right upper lobe

**Fig. 2:**
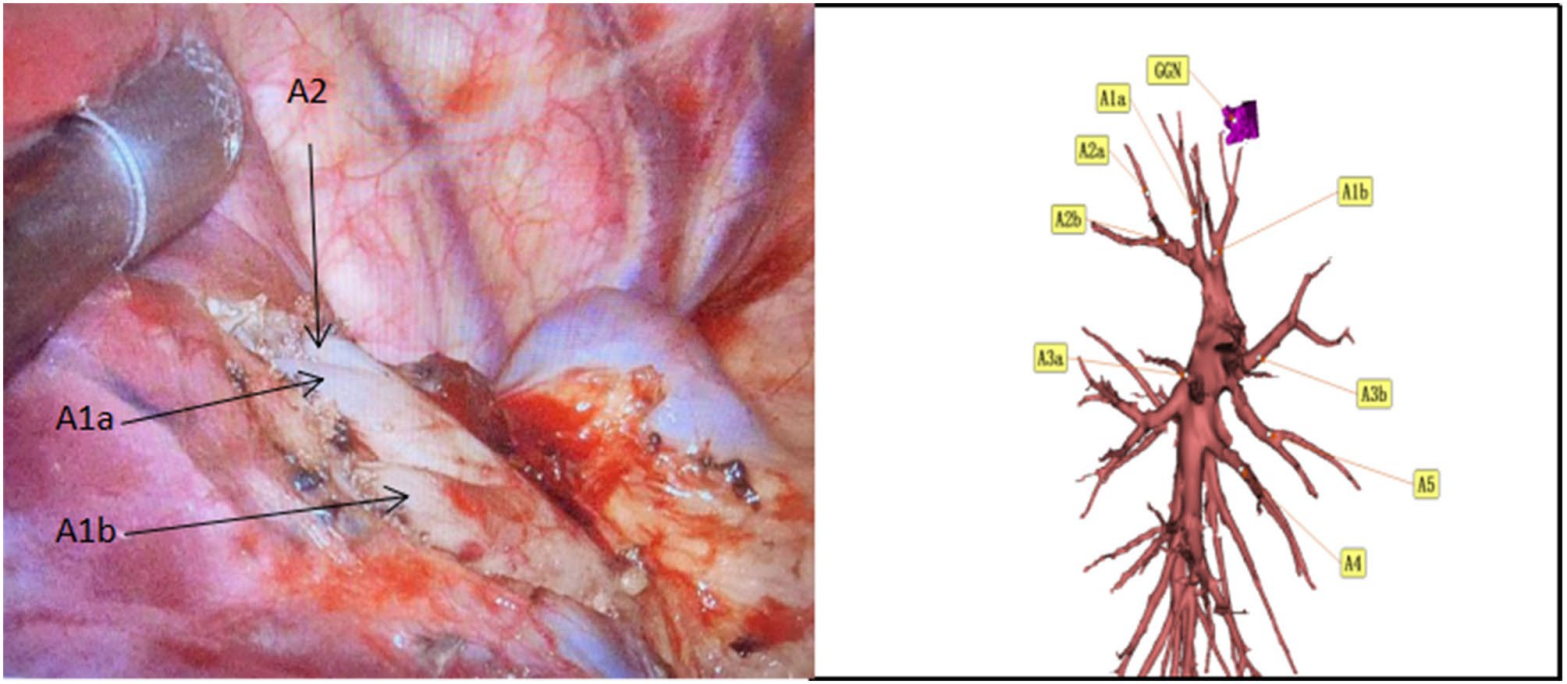
After dissecting the upper hilar pleura, we isolated the apical subsegmental artery (A1a) and anterior subsegmental artery (A1b). However, we did not observe the recurrent artery (Rec.A2 )

**Fig. 3:**
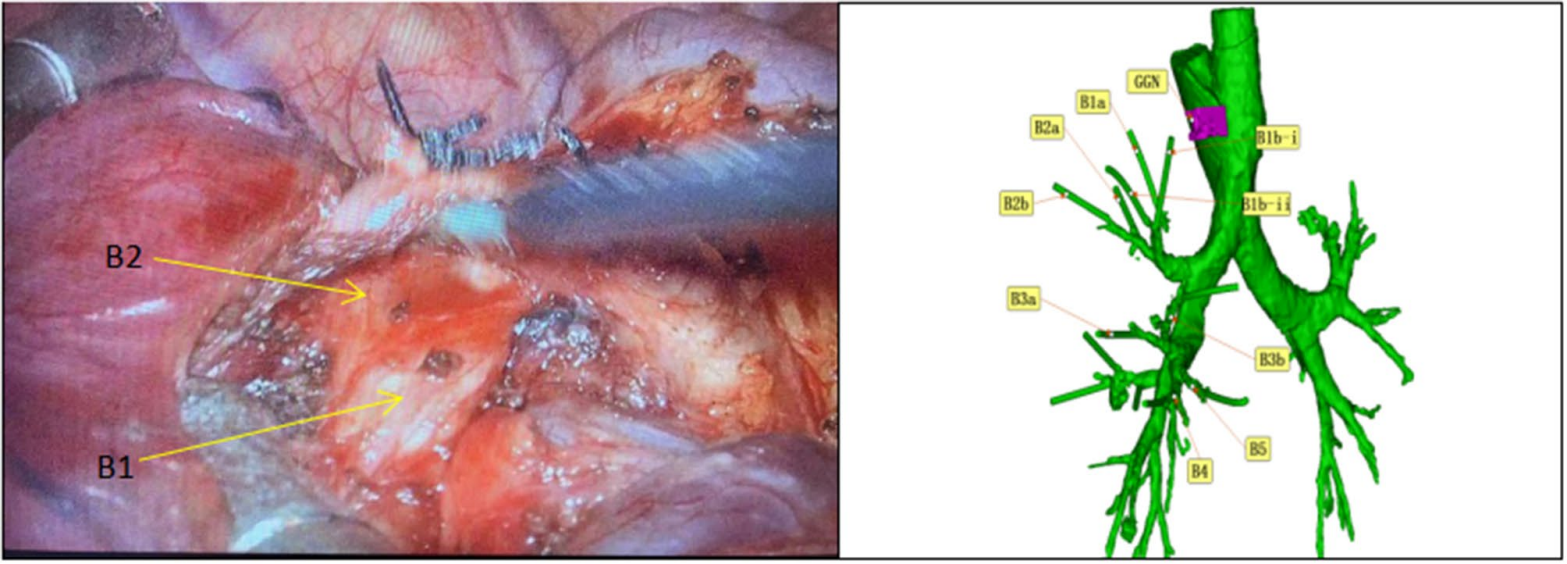
Following the resection of the arteries and peribronchial lymph nodes, a three-dimensional reconstruction technique was employed to visualize the apical segmental bronchus (B1) and posterior segmental bronchus (B2). However, the anterior bronchus (B3) was not located

**Fig. 4:**
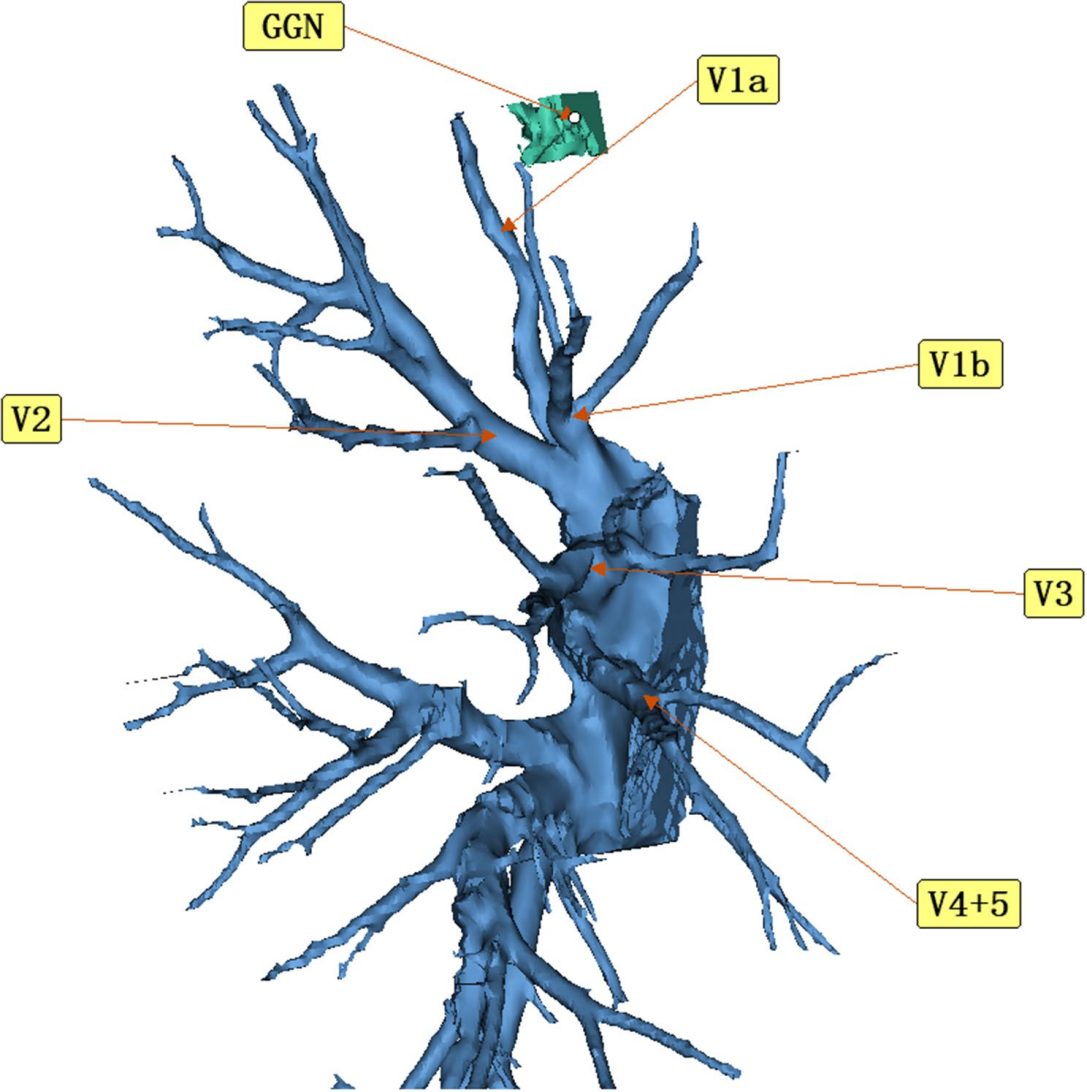
According to the three-dimensional reconstruction, it can be observed that the apical segmental vein (V1) and the posterior segmental vein (V2) merge into the right pulmonary vein, while the anterior segmental vein (V3) drains solely into the right pulmonary vein

**Fig. 5:**
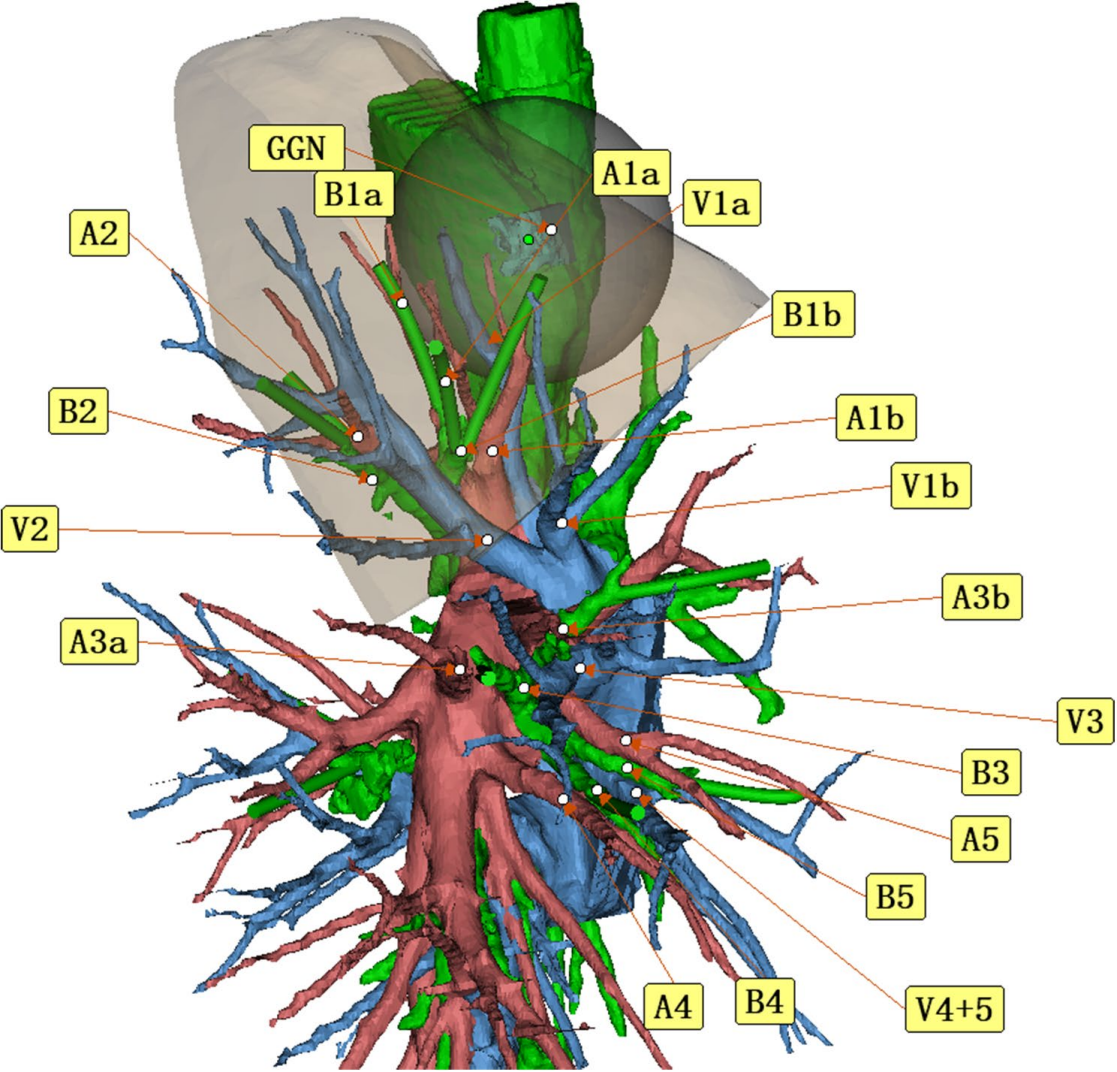
Utilizing three-dimensional reconstruction technology, it is evident that the nodule is situated in the S1 region, with an ample margin available for a safe S1 resection

A 47-year-old woman discovered a ground glass nodule(GGN) measuring approximately 8 mm in the right upper lobe(RUL) during a chest CT scan at our hospital over 3 years ago (Figs. 1, 2, 3, 4, 5). Throughout regular follow-up, no significant changes were observed in the nodule located in the right upper lobe. On July 19, 2023, a subsequent chest CT scan revealed that the nodule had increased in size by 10 mm and was hospitalized in our hospital on August 13,2023. Three-dimensional reconstruction of the chest prior to the operation displayed various variations in the bronchus and blood vessels: (1)the anterior bronchus originated from the right middle lung bronchus; (2) A3b and A5 shared a trunk; (3) A3a originated from the right inferior pulmonary trunk; (4) A1a and A2 shared a trunk; (5) A4 originated from the right basilar artery;(6)the right upper pulmonary arteries shared a trunk without a posterior ascending artery ; (7) The apical segmental vein and posterior segmental vein merge into the right pulmonary vein, while the anterior segmental vein solely drains into the right pulmonary vein. Based on the three-dimensional reconstruction, the nodule was identified in apical segmental(S1), and after conducting relevant examinations, the contraindication for surgery was ruled out.

S1 Segmentectomy of the right upper lung using thoracoscopic approach was accurately executed following thorough preoperative planning based on chest reconstruction in three dimensions. Initially, the pleura and hilum of the anterior mediastinum were incised, and the sections A1a and A1b were dissected and extracted. No branch of the anterior segmental artery (A3) was detected within the first branch of the right pulmonary artery trunk. Subsequently, separation of the first branch of the right lung trachea was conducted, and identification of the apical segmental bronchus (B1) and posterior segmental bronchus (B2) occurred, while B3 was not located. B1 was liberated and excised. Ultimately, the apical segmental of the right upper lung was successfully excised, and intraoperative analysis of frozen tissue confirmed the presence of micro-invasive adenocarcinoma, while sampling of lymph nodes was performed. The patient experienced an uneventful recovery and was discharged on the fifth day postoperatively. The postoperative histopathological examination confirmed the diagnosis of minimal invasive adenocarcinoma of the lung with no involvement of the surrounding lymph nodes.

## Discussion

The publication of the JCOG0802/WJOG4607L study has brought increased attention to anatomical segmentectomy for early lung cancer [1]. This surgical procedure involves the complete removal of the tumor with a sufficient surgical margin, while preserving as much normal lung tissue as possible [2]. Recent comparative studies on segmentectomy have highlighted the benefits of preoperative three-dimensional reconstruction technology [3–5]. This technology allows for the identification of variations in the trachea and blood vessels, enabling better preoperative planning and reducing the risk of complications. During the operation, it may be necessary to remove additional lung tissue because of cutting more blood vessels or trachea; Or cut less blood vessels or trachea without removing nodules or insufficient surgical margins.Additionally, there have been reports of rare variations in trachea and blood vessels in certain cases [6, 7]. Zhang et al. [7] reported variations in the bronchus and veins of the right upper lung in a patient who underwent VATS S1 + S2a segmentectomy. Zhang et al. [7] reported anomalous bronchi and pulmonary vessels in a patient who underwent thoracoscopic right posterior segmentectomy. In this study, we utilized a mimics 3D reconstruction technique to reconstruct the trachea and blood vessels prior to the operation. Through this technique, we were able to identify the abnormal trachea (B3 originating from the right middle lung bronchus) and blood vessels (A1a and A2 being common; A3b and A5 shared a trunk; A3a originating from the right inferior pulmonary trunk; A4 originating from the right basilar artery trunk). When performing VATS segmentectomy on patients with such abnormalities and relatively rare cases, it is crucial to conduct a correct preoperative evaluation. Failure to do so may increase the risk of surgery.Although the variant trachea and blood vessels belong to S3, and we performed a segmentectomy of S1, we confirmed the accuracy of the three-dimensional reconstruction during the operation, and further explained the importance of three-dimensional reconstruction for segmentectomy.

Preoperative three-dimensional reconstruction can confirm the variation of bronchi and pulmonary veins in the targeted lung segment. This allows for a detailed preoperative plan and accurate and safe performance of thoracoscopic segmentectomy under the guidance of three-dimensional reconstruction.

## Data Availability

All data generated or analysed during this study are included in this article.

## Abbreviations

3D: three dimensions
A1a: apical subsegmental artery
A1b: anterior subsegmental artery
A2: posterior segmental artery
A3: anterior segmental artery
A3b: medial subsegmental artery
A3a: lateral subsegmental artery
A4: lateral segmental artery
A5: medial segmental artery
B1: apical segmental bronchus
B2: posterior segmental bronchus
B3: anterior segmental bronchus
CT: computed tomography
GGN: ground glass nodule
RUL: right upper lobe
S1: apical segmental
S2a: posterior subsegment
VATS: Video assisted thoracic surgery
